# Lethal and sublethal effects of essential oil of *Lippia sidoides* (Verbenaceae) and monoterpenes on Chagas’ disease vector *Rhodnius prolixus*

**DOI:** 10.1590/0074-02760160388

**Published:** 2016-11-21

**Authors:** Marcela B Figueiredo, Geovany A Gomes, Jayme M Santangelo, Emerson G Pontes, Patricia Azambuja, Elói S Garcia, Mário G de Carvalho

**Affiliations:** 1 Universidade Federal Rural do Rio de Janeiro, Instituto de Ciências Exatas, Departamento de Química, Seropédica, RJ, Brasil; 2 Fundação Oswaldo Cruz, Instituto Oswaldo Cruz, Laboratório de Bioquímica e Fisiologia de Insetos, Rio de Janeiro, RJ, Brasil; 3 Universidade Federal Rural do Rio de Janeiro, Instituto de Florestas, Departamento de Ciências Ambientais, Seropédica, RJ, Brasil; 4 Universidade Estadual Vale do Acaraú, Centro de Ciências Exatas e Tecnologia, Sobral, CE, Brasil

**Keywords:** Rhodnius prolixus, natural compounds, biological activity, Lippia sidoides

## Abstract

The aim of this study was to identify the composition of the essential oil from leaves of *Lippia sidoides* (EOLS), a typical shrub commonly found in the dry northeast of Brazil, popularly known as “alecrim-pimenta”. Additionally, we investigated the nymphicidal, ovicidal, phagoinhibitory and excretion effects of EOLS, its major constituent thymol and its isomer carvacrol, on fourth instar nymphs and eggs of *Rhodnius prolixus*, the Chagas’ disease vector. The nymphicidal and ovicidal activity of thymol, carvacrol, and EOLS was assessed by tests using impregnated Petri dishes. The lethal concentration values (LC50) for EOLS, carvacrol, and thymol were 54.48, 32.98, and 9.38 mg/cm^2^, respectively. The ovicidal test showed that both carvacrol and thymol (50 mg/cm^2^) inhibited hatching (50% and 23.3%, respectively), while treatments with 10 mg/cm^2^ or 50 mg/cm^2^ EOLS did not affect the hatching rate at all (80% and 90%, respectively). We observed an anti-feeding effect in insects fed with blood containing natural products at the higher concentrations (100 µg/mL). Finally, excretion rate was affected by EOLS and carvacrol, but not by thymol. These findings offer novel insights into basic physiological processes that make the tested natural compounds interesting candidates for new types of insecticides.

*Rhodnius prolixus* (Stal, 1859) is a haematophagous insect belonging to family Reduviidae, subfamily Triatominae. These insects are vectors of the hemoflagellate protozoan parasite *Trypanosoma cruzi*, the etiological agent of Chagas disease, also known as American trypanosomiasis. The *R. prolixus* distribution range includes Central America and the northern South America (Rey 2008). Chagas disease is an endemic disease in large areas of South and Central America and an important health problem in these areas. Chagas disease affects nearly six million people worldwide, mostly in Latin America. Chronic Chagas disease infection is responsible for the death of approximately 14,000 people annually, while acute infection is asymptomatic. Hitherto, there is neither any preventative vaccine nor any treatment against the chronic phase of Chagas disease. In this context, the development of methods aiming to eliminate the Chagas disease vector through insecticides is one of the best strategies to prevent the disease. However, the continuous use of synthetic insecticides like pyrethroids and deltametrin leads to resistance of the target organisms and environmental pollution (Corrêa & Salgado 2011).

Some natural products synthesised by plants as secondary metabolites are an efficient alternative to synthetic insecticides (Corrêa & Salgado 2011). These compounds are known to exhibit biocidal properties and have been used as extracts, pure allelochemicals, or essential oils. They are economically viable and biodegradable and, in some cases, display high activity against several vector insects (Gomes et al. 2012). In addition, these compounds exhibit low toxicity against mammals, with high selectivity and low phytotoxicity (Corrêa & Salgado 2011). In several insect orders, treatment with essential oils leads to larvicide and adulticide. In addition, essential oils affect the development of insects and may interfere with the moulting process, mating behaviour, and oviposition (Tripathi et al. 2009), but few natural compounds act as fagoinhibitory substances.

The botanical family Verbenaceae comprises approximately 175 genera distributed principally in the Southern Hemisphere. The genus *Lippia* contains about 200 species distributed throughout Central and South America and tropical Africa (Terblanché & Kornelius 1996). The species *Lippia sidoides* Cham, popularly known in Brazil as ‘alecrim-pimenta’, is a typical shrub commonly found in the semi-arid northeast of Brazil. Several studies have reported the broad range of *L. sidoides* essential oil biological activity including antimicrobial (Botelho et al. 2007), acaricidal (Gomes et al. 2012), and insecticidal (Lima et al. 2013) effects. Commonly, the major compounds of *L. sidoides* essential oil are the monotherpene thymol and its isomer carvacrol (Terblanché & Kornelius 1996). Both substances have a powerful activity against several invertebrate animals.

The present study aimed to describe the composition of the essential oil from *L. sidoides* leaves (EOLS). Additionally, we describe the biological activity of EOLS and its major constituent, thymol, as well as the thymol isomer carvacrol, against fourth instar nymphs of *R. prolixus*. Specifically, we evaluated the nymphicidal, ovicidal, excretion and fagoinhibitory effects of EOLS, thymol and carvacrol at different concentrations.

## MATERIALS AND METHODS

*Essential oils and monoterpenes* - EOLS was acquired from Natural Products LTDA (PRONAT - Horizonte, CE, Brazil). This company cultivates the plant and performs the extraction of its oil by the method of distillation by steam distillation. The monoterpenes carvacrol (98%) and thymol (100%) were obtained from Sigma-Aldrich^®^ companies (São Paulo, SP, Brazil) and Vetec Química Fina LTDA (Duque de Caxias, RJ, Brazil), respectively.

*Analysis of the essential oil* - The chemical composition of the essential oil was analysed in a gas chromatograph coupled to a mass spectrometer (GC/MS - Shimadzu QP-2010 Plus), equipped with a *Factor Four/VF-5ms* fused-silica capillary column (30 m x 0.25 mm x 0.25 µM film thickness), using helium as carrier gas at 1 mL/min. The initial oven temperature was 35ºC. After held constant for 2 min, temperature was increased at a rate of 4ºC min^-1^ to 180ºC, followed by 10ºC min^-1^ to 250ºC, with a final isotherm (250ºC) for 20 min. The sample injection volume was 1 µL (1:50 split mode). The injector and detector temperatures were both 250ºC. The mass spectra were obtained in a range of *m/z*10 - 300, by the electron impact technique (MSEI) at 70 eV. The quantitative analysis of the chemical composition of the oil was carried out in a gas chromatograph coupled to an HP 5890 Series II flame ionization detector (FID), using the same operational conditions and the same type of column as in the GC/MS analysis, with exception of the injector and detector temperatures that were of 240 and 300ºC, respectively. The injector and detector temperatures were 240 and 300ºC, respectively. The percentage of each constituent was calculated by the integral area under the respective peaks in relation to the total area of all the sample constituents.

The identified chemical constituents in the essential oil were detected by visual comparison of their mass spectra with those in the literature and spectra supplied by the equipment database (NIST08), as well as by comparison of the retention indices with those in the literature (Adams 2007). A standard solution of n-alkanes (C8-C20) was injected under the same chromatographic conditions as the sample and used to obtain the retention indices. The identification of the major constituents was done based on the information obtained from the mentioned analytic methods, together with the data generated by comparison of the nuclear magnetic resonance spectra of hydrogen (NMR ^1^H, in a Bruker 500 MHz spectrometer) of the oil, standard pure thymol (Vetec Química Final Ltda, Rio de Janeiro, RJ, Brazil) and a mixture of oil and thymol.

*Insects* - In order to verify the effects of natural compounds in the insect biology we used fourth instar *R. prolixus* nymphs and eggs throughout the study. After molting, insects were starved for 15-20 days and were randomly chosen when required. All insects were raised and maintained in a laboratory colony and fed on defibrinated rabbit blood through a membrane feeding apparatus.

*Nymphcidal activity: nymphs on impregnate Petri dishes* - Preliminary essays were performed in order to choose the adequate concentrations to obtain a dose-response relationship. Based on these results, a second experiment was performed to estimate the LC_50_ values. Five different concentrations of EOLS (50, 52, 55, 58 and 60 mg/cm^2^), and four different concentrations of carvacrol (20, 30, 40 and 50 mg/cm^2^) and thymol (1, 5, 8 and 10 mg/cm^2^) were tested. Experiments were replicated three times and each test comprised two replicates for each concentration.

*Ovicidal activity*: *eggs on impregnate Petri dishes* - The ovicidal activity of EOLS, thymol and carvacrol was performed using 9 cm diameter glass Petri dishes. The method proposed by Laurent et al. (1997) was adapted for this essay. After preliminary essays, the Petri dishes were directly treated with 1 mL EOLS, thymol and carvacrol solutions diluted in 1 mL of acetone in final concentrations of 10 or 50 mg/cm^2^ of each compound. Control dishes were treated only with acetone or untreated at all. The dishes were covered with glass lids and placed on a chamber with a constant temperature (27ºC ± 2) and humidity (65% ± 5). The dishes were dried at room temperature for 15 min.

The eggs of *R. prolixus* were collected five days after oviposition, selected according to the characteristic red colour. At five days old, the embryo displays an appearance of head and thoracic appendages with the abdomen quite unsegmented (Mellanby 1935). Ten eggs were placed inside each dish, getting directly in contact with the treated surface of Petri dishes and exposed to EOLS, thymol and carvacrol during 15 days. The percentage of the eggs that hatched was determined every day until the 15th day. The hatching experiment was carried out three times in different days with two Petri dishes for each compound and concentration.

*Oral treatment, excretion and antifeedant activity* - For the oral treatment of insects, the compounds EOLS, thymol and carvacrol were diluted in hydroalcoholic solution (1:2 ethanol-saline) just before the feeding assay. Different concentrations of these compounds were added to the blood meal with final concentrations of 10, 50 and 100 µg/mL of blood. Groups of 4th-instar larvae were allowed to feed through a membrane feeding apparatus. A control group was fed with blood containing only hydroalcoholic solution. Blood intake was determined by body weight differences obtained right after and just before feeding. The excretion after 24 h was determined by body weight differences obtained right after feeding and 24 h later. Because the excretion process is also affected by the volume of ingested blood, we calculated excretion rates through the formula: weight of excretion / weight of blood intake.

*Statistical analyses* - Different natural products were used as predictive variables and ingested blood, excretion rates and the proportion of hatched eggs as the response variables. The effect of the different natural products was compared with analysis of variance (ANOVA). If significant differences among values were observed, Tukey post hoc tests were applied between pairs of values. The requirements of normal distribution and homogeneity of variances were checked using Shapiro-Wilks and Bartlett tests, respectively. Before analysis, an arcsine square root transformation was applied for the excretion rates and the proportion of hatched eggs. All analyses were carried out with Prism 5.0 statistical software. Finally, LC_50_ values and 95% confidence intervals (95% CI) were calculated using the probit methods (Finney 1971). LC_50_ values of the three compounds were considered statistically different if the 95% CI did not overlap.

## RESULTS

Twenty-two substances were identified, accounting for 98.47% of the essential oil. Their retention indices in the *Factor Four/VF-5ms* column and percent composition are listed in Table I. Of these, there were nine monoterpene hydrocarbons (21.01%), eight oxygenated monoterpenes (72.21%), four sesquiterpene hydrocarbons (4.70%) and one oxygenated sesquiterpene (0.55%).

**TABLE I:** Chemical composition, calculated retention index (RIC), percentages of identified components (%) and classes of the same in the essential oil of *Lippia sidoides*

Compounds	RI_c_	(%)
Monoterpene hydrocarbons		21.01
α-Thujene	930	0.16
α-Pinene	938	0.74
β-Pinene	986	0.12
Myrcene	994	3.57
δ-(3)-Carene	1012	0.17
α-Terpinene	1022	0.78
*o*-Cymene	1035	14.84
*E-*β*-*Ocimene	1052	0.04
g-Terpinene	1065	0.59
		
Oxygenatedmonoterpenes		72.21
6,7-Epoxymyrcene	1095	0.20
Linalool	1102	0.23
Ipsdienol	1164	0.12
Terpinen-4-ol	1188	0.16
*p*-Cymen-8-ol	1198	0.26
a-Terpineol	1202	0.49
T*hymolmethylether*	1236	0.84
T*hymol*	1315	69.91
		
Sesquiterpenehydrocarbons		4.70
α-Ylangene	1385	0.11
*E-*Caryophyllene	1433	4.04
α-Humulene	1467	0.21
Viridiflorene	1487	0.34
		
Oxygenatedsesquiterpene		0.55
Caryophyllene oxide	1597	0.55

Total		98.47

*Nymphicidal assay: contact surface* - In order to establish the LC of EOLS, thymol and carvacrol, nymphicidal experiments using contact surface assay were performed. The LC_50_ values for EOLS, thymol and carvacrol were 54.48, 9.38 and 32.98 mg/cm^2^, respectively. Since the confidence intervals of the different compounds do not overlap, the LC_50_ values of EOLS, thymol and carvacrol are significantly different (Table II). Based on these data, it is possible to verify the best performance of thymol on nymphs of *R. prolixus*, followed by carvacrol.

**TABLE II:** Lethal concentration 50 (LC50) and 95% confidence intervals (95% CI) of essential oil from *Lippia sidoides* (EOLS), thymol and carvacrol to *Rhodnius prolixus* 4th instar-nymphs. Different letters following LC50 values indicate significant differences among compounds since the 95% CIs do not overlap

Compound	LC_50_ (mg/cm^2^)	95% CI
EOLS	54.48^a^	52.19-56.87
Thymol	9.38^b^	7.88-11.17
Carvacrol	32.98^c^	28.98-38.38

**Fig. 1:**
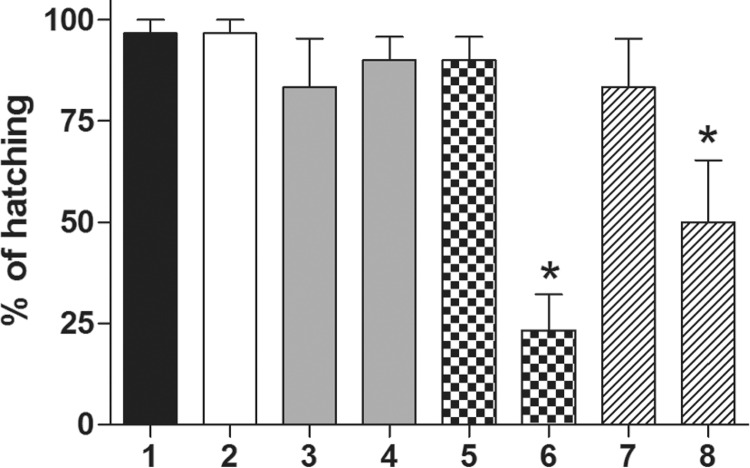
: mean (+1SE) percentage of hatching of *Rhodnius prolixus* eggs 15 days after exposure to essential oil and monotherpenes. Control (1); acetone 100% (2); EOLS 10 mg/cm2 (3); EOLS 50 mg/cm2 (4); thymol 10 mg/cm2 (5); thymol 50 mg/cm2 (6); carvacrol 10 mg/cm2 (7); carvacrol 50 mg/cm2 (8). *: designates significantly different values when compared to control (p < 0.05).

*Ovicidal assay: contact assay* - Considering all treatments, the mean hatching rate varied from 23.3-96.7% (Fig. 1) and differed between treatments (ANOVA, *F* = 7.96, p = 0.0003). The mean hatching rate of eggs exposed to 10 mg/cm^2^ or 50 mg/cm^2^ of EOLS were 80% and 90%, respectively, and did not differ from that observed in control treatments (96.7%). When exposed to thymol, the hatching rates were 90% and 23.3% under 10 mg/cm^2^ and 50 mg/cm^2^, respectively. However, only the higher concentration of 50 mg/cm^2^ of thymol affected the hatching rate when compared to control eggs. Similar results were observed for the carvacrol. Hatching rates when exposed to carvacrol were 83.3% and 50% under 10 mg/cm^2^ and 50 mg/cm^2^, respectively. Only the higher concentration of 50 mg/cm^2^ of carvacrol affected the hatching rate. Based on these results, we can infer that the hatching rate of eggs is considerably inhibited after 15 days of incubation with 50 mg/cm^2^ of thymol (76.7% of inhibition), followed by 50 mg/cm^2^ of carvacrol (50% of inhibition).

**Fig. 2:**
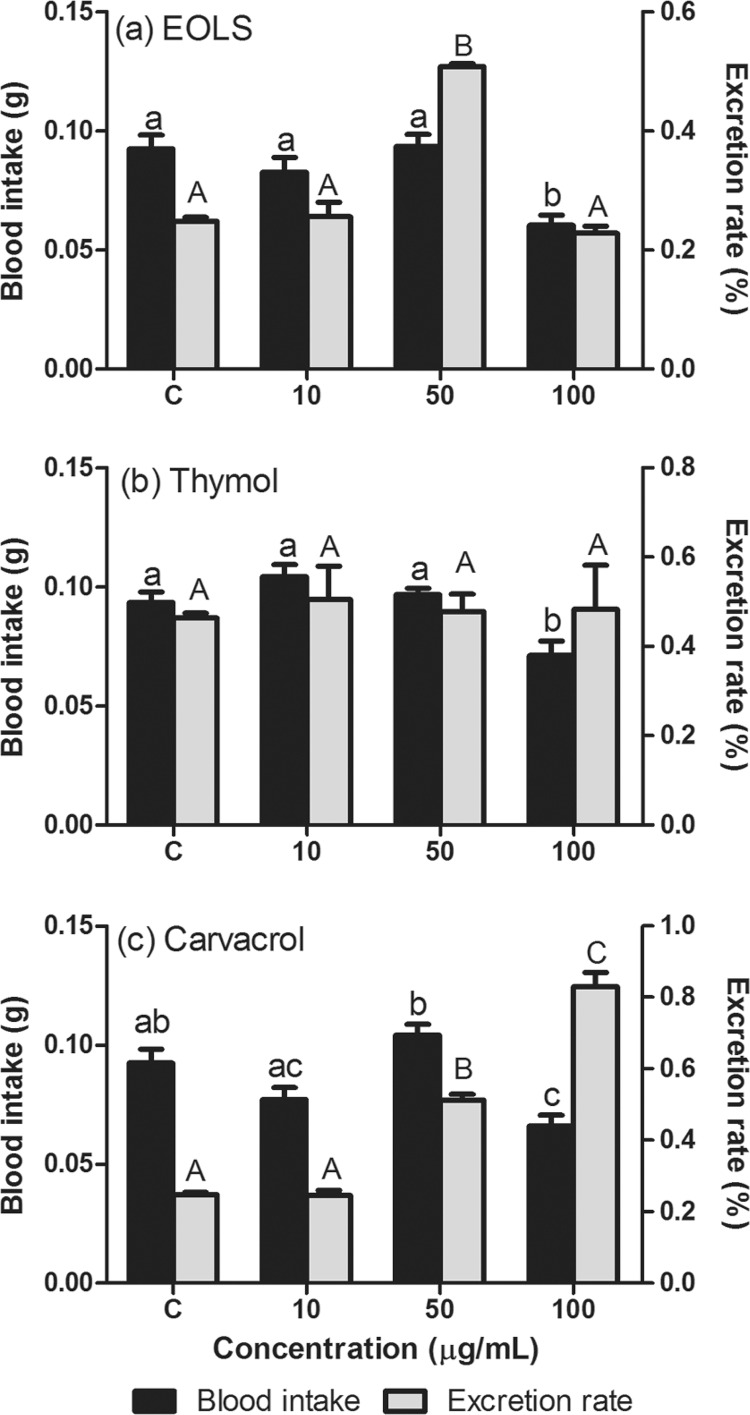
: mean (+1SE) blood intake and excretion rate (24 h after feeding) of *Rhodnius prolixus* fourth instar nymphs orally treated with essential oil of *Lippia sidoides* (EOLS) (a), thymol (b), and carvacrol (c). Different lowercase letters above the black bars denote significant differences in blood intake between treatments. Different capital letters above the grey bars denote significant differences in excretion rates between treatments.

*Excretion and antifeedant activity* - The blood intake was affected by the presence of EOLS (ANOVA, *F* = 8.64, p = 0.0002), thymol (ANOVA, *F* = 8.54, p = 0.0002) and carvacrol (ANOVA, *F* = 12.78, p < 0.0001). However, the effect of each compound depended on the concentration used. For example, we observed no antifeedant effect in groups fed with blood containing 10 or 50 µg/mL of EOLS or thymol, since there were no differences between the blood intake of treated and control groups (p > 0.05) (Fig. 2A-B). On the other hand, the treatment with 100 µg/mL of EOLS or thymol resulted in a decrease of blood intake by nymphs when compared to the other groups (p < 0.05). Similarly to the previous compounds, the 100 µg/mL of carvacrol treatment also reduced the blood intake when compared to control organisms (Fig. 2C). However, the 50 µg/mL of carvacrol treatment also differed from 10 µg/mL.

The excretion rate of insects was also affected by EOLS (ANOVA, *F* = 58.78, p < 0.0001) and carvacrol (ANOVA, *F* = 124.9, p < 0.0001), but not by thymol (ANOVA, *F* = 0.09, p = 0.96) (Fig. 2). The organisms treated with 50 µg/mL of EOLS displayed higher excretion rates when compared to the remaining treatments (Fig. 2A). The treatments with carvacrol resulted in higher excretion rates at the groups orally fed with 50 µg/mL and 100 µg/mL when compared to 10 µg/mL and control groups (Fig. 2C).

## DISCUSSION

*Essential oil composition* - The main components of the EOLS analysed in this study were thymol (69.91%), *o*-cimene (14.84%), *E-*caryophyllene (4.04%), and myrcene (3.57%), corroborating an earlier study (Camurça-Vasconcelos et al. 2007). In addition, according to the literature, the percentage of thymol varies between 2.30 and 84.9% in EOLS (Botelho et al. 2007, Gomes et al. 2012). On the other hand, the chemical analysis of the EOLS did not detect significant percentages of carvacrol, a thymol isomer that is usually described in the literature as the most abundant component of EOLS, along with its isomer (Botelho et al. 2007). Few studies report the absence of carvacrol in the chemical composition of EOLS (Camurça-Vasconcelos et al. 2007). Several factors may explain such absence, because the chemical composition of EOLS are affected by factors such as climate, soil composition, plant age, and stage of the growth cycle, as well as the method used to obtain the oil.

*Nymphicidal activity* - In this study, using the contact surface test, we observed a nymphicidal effect of EOLS against *R. prolixus* nymphs with an LC_50_ of 54.48 mg/cm^2^ after 24 h of exposure. Despite the lack of literature about the insecticide activity of EOLS in triatomines, some studies report high levels of repellent activity in adults of *R. prolixus* caused by the essential oil of *Thymus zygis* (Lamiaceae), which contains a high percentage of thymol (74%) (Sainz et al. 2012). The monoterpene thymol, EOLS major component (69,91%), presents high levels of toxicity against several insects (Pavela 2011). In triatomines, recent studies described that the treatment of *Triatoma infestans* and *R. prolixus* with thymol caused hyperactivity and knock-down effects in first nymphs while the repellency effect was observed only in *T. infestans* nymphs (Moretti et al. 2013).

We observed a nymphicidal effect 24 h after *R. prolixus* nymphs were exposed to the essential oil, with an LC_50_ of 54.48 mg/cm^2^. Additionally, the pure monoterpene thymol caused a deleterious effect against *R. prolixus* nymphs under lower concentrations, reaching a LC_50_ of 9.38 µg/cm^2^. This difference suggests that the phenolic compounds are more toxic to the triatomine than EOLS (Table II). We also observed a considerable activity of carvacrol against *R. prolixus*, presenting a LC_50_ of 32.98 mg/cm^2^, which is more potent than EOLS. However, the contact surface treatment with carvacrol resulted in lower mortality of *R. prolixus* nymphs when compared with its isomer thymol. The higher insecticide activity of thymol in comparison to carvacrol is widely described in the literature (Singh et al. 2009). In triatomines, the exposure of *R. prolixus* and *T. infestans* nymphs to carvacrol causes an increase in locomotor activity, repellency and knock-down effect (Moretti et al. 2013).

These results suggest that in *Rhodnius*, the treatment with the monoterpenes thymol or carvacrol alone is more efficient than the treatment with the mixture of different monoterpenes and sesquiterpenes found in EOLS chemical composition. Our results showed the greater nymphicidal activity of the monoterpenes thymol and carvacrol in nymphs of *R. prolixus.* In fact, our exposure tests showed that the nymphicidal activity of thymol was 5.8 times more lethal than EOLS and 3.5 times more lethal than carvacrol. We also observed that the treatment of the nymphs with carvacrol was 1.7 times more lethal than EOLS. Indeed, plants in which the phenolic compounds thymol and carvacrol are plentiful display a greater insecticide effect (Karpouhtsis 1998). Several studies indicate a close association between the insecticide activities of thymol and carvacrol and the ability to block the gamma - aminobutyric acid (GABA) and/or octopaminergics systems of arthropod (Rattan 2010).

The blockage of the GABA chloride gated channel leads to a disorder in the physiological and immunological systems causing convulsion and central nervous hyper-excitation, culminating with insect death (Rattan 2010). Regarding the octopaminergic system, several studies already described high levels of octopamine (octopaminergic system neurotransmitter) in the central and peripheral nervous systems of most invertebrate species, especially in insects (Farooqui 2011). In these organisms, the neurotransmitter octopamine plays several roles, acting as a neuromodulator and neurohormone. In some cases, under stressful conditions, this neurotransmitter is released in the hemolymph of the insects playing a neurohormonal role (Farooqui 2011). Generally, the octopamine interact with two classes of receptors (octopamine-1 and octopamine-2) and the interruption of these interaction results in a total breakdown of the insects nervous system. Therefore, the octopaminergic system represents an efficient target in insect control (Tripathi et al. 2009).

*Ovicidal activity* - The ovicidal property of essential oils against Chagas disease vectors eggs are well described in the literature. Several essential oils obtained from different plant species, such as *Eryngium* sp. (Apiaceae), *Tagetes pusilla* and *T. minuta* (Asteraceae), decrease the hatching rate of eggs during exposure tests (Laurent et al. 1997). However, we found that the treatment with EOLS was not effective in inhibiting *R. prolixus* eggs. This result differs from the ovicidal tests performed in different species of arthropods (Maciel et al. 2009, Lima et al. 2013). In addition, the literature describes that their essential oils pure constituents may also have ovicidal effects against triatomines. For example, among 12 constituents of essential oils tested, the sesquiterpene *E*-nerolidol showed higher ovicidal activity against *T. infestans* eggs (Laurent et al. 1997). In this study, only thymol and carvacrol compounds were able to inhibit egg hatching. We observed a decrease in hatching, only in eggs treated with 50 mg/cm^2^ of thymol or carvacrol. The ovicidal effect of thymol against other invertebrate eggs has been described in several studies (Camurça-Vasconcelos et al. 2007). Similarly to our results, the exposure of eggs to the monoterpene thymol was more active against *Chilo partellus* (Lepidoptera: Pyralidae) when compared to its isomer carvacrol (Singh et al. 2011).

Because in insects the eggs are formed by many layers, the embryo may survive even during long and adverse periods. However, lipophilic substances, such as the essential oil constituents, are able to harden the eggs membrane, preventing the hatching process by interfering with the water balance and gas exchange (Lima et al. 2013).

When observing our results of nymphicidal and ovicidal activity, despite the differences in mobility and time of exposure, we may suggest that the fourth stage nymphs are more susceptible to the natural compounds in comparison to the eggs of *R. prolixus*. Similar results were described for *T. infestans* nymphs and *Lutzomia longipalpis* larvae, which were more sensitive than eggs to natural compounds (Laurent et al. 1997, Maciel et al. 2009). Although the neurotoxic properties of monoterpenes are widely described in the literature, the toxicity of these substances becomes more evident when the insect embryo nervous system begins to develop. The lower permeability of the eggs outer surface in early embryogenesis could be another factor that may explain the greater tolerance of eggs against the phytochemicals evaluated in our study (Maciel et al. 2009). Hitherto, no studies had shown that thymol and carvacrol are able to inhibit the hatching of *R. prolixus* eggs.

*Excretion and antifeedant activity* - Usually, the mode of action of antifeedant compounds involves the insects taste cells, stimulating specific receptors that send a negative signal to the feeding centre in the central nervous system of the insects. The feeding process has a central role in the triatomines life cycle since the blood meal will trigger a series of physiological and immunological reactions essential for survival of these insects. The antifeedant effect may occur by two mechanisms, through chemoreception - primary antifeedant effect - or through an internal feedback mechanism - secondary antifeedant effect (Dubey 2011).

According to the literature, the secondary metabolites with antifeedant action against a broad range of insects includes essential oils from various types of plants (Singh et al. 2011). In relation to monoterpenes, several studies describe the anti-feeding properties of terpenoids, sesquiterpenes and monoterpenes (Tripathi et al. 2009). However, few studies report the fagoinhibition effects of essential oils and terpenes against triatomines. Mello et al. (2007) observed that the orally treatment of *R. prolixus* nymphs with the essential oil of *Pilocarpus spicatus* (Rutaceae) decreased significantly the blood intake in comparison to the control group. Some antifeedant compounds interfere with the perception of feeding stimulants preventing the insects from obtaining the correct taste information, affecting the insect feeding behavior (Garcia et al. 1991). However, in our case, the antifeedant effect of carvacrol and thymol against *Rhodnius* were not strongly enough since only the highest dose of these two compounds led to a decrease in the blood intake when compared to the control group.

Our results suggest that the oral treatment with the natural compounds interfere in the blood intake, but only the higher concentration (100 µg/mL) was efficient in antifeedant activity. Perhaps, the insect digestive system may efficiently metabolise low concentrations of monoterpenes and the essential oil substances before reaching the hemolymph, the central nervous system or other insect target organs.

The excretion process plays a central role in the physiology and homeostasis maintenance of insects, since the rapid elimination of fluids with high levels of sodium just after feeding prevents the hemolymph dilution and enables the concentration of nutritious parts of the blood meal, essential for insect development (Maddrell 1963). The abdominal distention caused by blood ingestion activate some receptors and induce the release of diuretic hormones from thoracic ganglion masses. These hormones stimulate the water and ion transport across the stomach wall to the hemolymph and Malphigian tubules increasing the urine secretion in 100 times, eliminating 47-76% of ingested amount few hours after blood feeding and reestablishing the osmotic and physiological equilibrium (Madrell et al. 1991).

In *R. prolixus*, the excretion process is affected by the volume of ingested blood and starvation period (Wigglesworth 1972). During the blood meal the nymphs ingest a volume of blood enough to increase their weight up 12 times. In this state, the insects are susceptible to predation; therefore they must quickly eliminate the excess of water and salt, through the excretion process, reducing its own volume (Maddrell 1963). Our results show that the excretion rates are affected by the ingestion of EOLS and carvacrol, but not by the ingestion of thymol. These results suggest that the various constituents of EOLS may have acted antagonistically on nymphs of *R. prolixus*. In some cases, the biological property of the essential oil can be given to synergistic or antagonistic action of its various constituents (Burt 2004, Bakkali et al. 2008). Maybe the blood feeding with the essential oil and the monoterpene carvacrol leads to an osmotic imbalance (up or down) affecting the modulation of insect physiology system. Additionally, since the excretion process is also directly related to the amount of blood meal, the similar excretion rates in control and 100 µg/mL of EOLS treatment are due to lower blood intake in the presence of high concentrations of the essential oil.

**Fig. 3:**
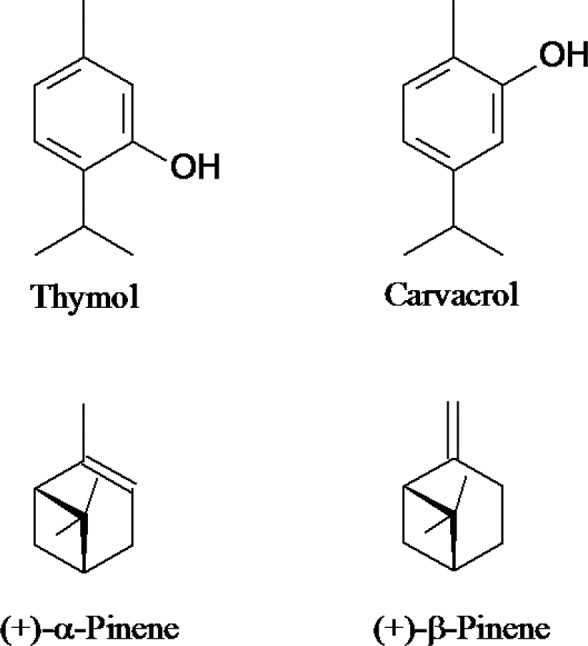
: different positions of hydroxyl groups in thymol and carvacrol structures.

As described in this study, thymol was more effective than carvacrol in relation to both ovicidal and larvicidal properties. High toxicity levels of carvacrol in comparison to thymol was also verified by Moretti et al. (2013), who evaluated the knock-down effect of these monoterpenes on nymphs of *T. infestans* first stage and *R. prolixus.* Although the structures of these phenolic compounds are similar, with the same chemical formula and molecular weight, they differ in the position of the hydroxyl group on the aromatic ring, so they are classified as position isomers (Fig. 3). The relative position of the hydroxyl group on the aromatic ring may have influenced the biocide potential of these monoterpenes against *R. prolixus* nymphs and eggs since the structural changes in monoterpenoids may lead to greater biological activity (Simas et al. 2004). The volatile properties of these compounds are extremely interesting since their steam can penetrate cracks, holes, bricks and wood hen houses (the main triatomines habitat), potentially affecting some physiological and life cycle parameters of *R. prolixus*. Further studies on synergistic effects between the essential oils monoterpenes are under consideration.
